# Probable aerosol transmission of SARS‐CoV‐2 in a poorly ventilated courtroom

**DOI:** 10.1111/ina.12866

**Published:** 2021-06-11

**Authors:** David Vernez, Sophie Schwarz, Jean‐Jacques Sauvain, Christiane Petignat, Guillaume Suarez

**Affiliations:** ^1^ Department of Occupational Health and Environment Center for Primary Care and Public Health (Unisanté University of Lausanne Lausanne Switzerland; ^2^ Public Health Service Vaud Canton Lausanne Switzerland

**Keywords:** aerosol transmission, COVID, infectious disease, modeling, ventilation

## Abstract

There is increasing evidence of SARS‐CoV‐2 transmission via aerosol; the number of cases of transmission via this route reported in the literature remains however limited. This study examines a case of clustering that occurred in a courtroom, in which 5 of the 10 participants were tested positive within days of the hearing. Ventilation loss rates and dispersion of fine aerosols were measured through CO_2_ injections and lactose aerosol generation. Emission rate and influencing parameters were then computed using a well‐mixed dispersion model. The emission rate from the index case was estimated at 130 quanta h^−1^ (interquartile (97–155 quanta h^−1^). Measured lactose concentrations in the room were found relatively homogenous (*n* = 8, mean 336 µg m^−3^, SD = 39 µg m^−3^). Air renewal was found to play an important role for event durations greater than 0.5 h and loss rate below 2–3 h^−1^. The estimated emission rate suggests a high viral load in the index case and/or a high SARS‐CoV‐2 infection coefficient. High probabilities of infection in similar indoor situations are related to unfavorable conditions of ventilation, emission rate, and event durations. Source emission control appears essential to reduce aerosolized infection in events lasting longer than 0.5 h.

**Practical implications**

- This case study contributes to the growing body evidence highlighting possible SARS‐CoV‐2 transmission through aerosol.
- The known exposure conditions in the courtroom allow us to reasonably exclude other transmission modes.
- Probabilities of transmission in room of similar sizes and various event durations and air renewal were estimated.

## 1 INTRODUCTION

The role of aerosols in the transmission of COVID is currently under debate. Superspreading events, in which aerosols may have been a significant factor, are regularly reported in the press. However, the cases reported in the scientific literature remain rare and we were able to identify few studies that explicitly attribute the emission to aerosols (see Table 1). In most of these cases, the evidence of aerosol contamination is also indirect, because contamination via surfaces or large droplets alone is not sufficient to explain the attack and reproduction rates observed.^1^ A similar observation was made after the systematic analysis of 318 outbreaks in China, where transmission in confined and poorly ventilated spaces was attributed to aerosols.^2^

**TABLE 1:** Reported case of spreading events with a strong suspicion of aerosols transmission

Location	Situation	Reference
Skagit Valley (USA)	53 of 61 choir members tested positive after a rehearsal of 2½ hours. The inferred mean emission rate was E = 970 (± 390 SD) quanta per hour.	^27^
Diamond Princess Cruise Ship	621 passengers out of 3711 were infected on board the cruise ship "Diamond Princess". The estimated reproduction rate for this confined case is about 11.	^31^
Zhejiang province (China)	24 out of 68 passengers were infected during a 100‐minute bus trip. No significant difference in the attack rate depending on the position in the bus, suggesting airborne contamination.	^33^
Wuhan (China)	A retrospective analysis of cases among health care staff showed that none of the 278 staff members using N95 masks were infected with SARS‐CoV−2, while 10 of the 213 doctors or nurses were infected.	^34^
Guangzhou (China)	10 out of 68 people present in the same room were infected after eating in a restaurant. The room was poorly ventilated due to a lack of outdoor air supply.	^32^
Seoul (South Korea)	97 out of 1143 call center employees were infected. 94 of them worked on the same floor of the building. The attack rate on this floor was 43%.	^35^

The term aerosol generally refers to exhaled respiratory particles <5–10 µm that are both likely to remain suspended in the air for a long time and to penetrate deeply into the respiratory system.^3^ Human activities such as breathing and speech produce exhaled respiratory particles mostly below the 5–10 μm range.^4^ Zhu reports that a healthy individual emits 10–10^4^ particles per liter of exhaled air, 95% of which are <1 μm aerosols.^5^ During speech, the emission can reach 5 × 10^3^ particles per minute. Coughing generates 10^3^–10^4^ particles ranging between 0.5 and 30 μm, a majority of them below 2 μm. A sneeze produces about 10^6^ particles between 0.5 and 16 μm.^6^, ^7^ The emissivity increases with the energy supplied (breathing <speech < cough), with a strong variability between test subjects.^7^

The sedimentation time of aerosols can reach several hours and their aeraulic behavior is close to that of a gas. Their displacement is essentially determined by the movement of the ambient air.^8^ However, the largest droplets, typically greater than 50–100 μm, will follow ballistic trajectories due to the force of gravity and their initial kinetic energy (e.g., in case of coughing). The sedimentation mechanism will dominate and the droplets will settle on the ground after a few seconds, or ten seconds.^8^ Between 10 and 50 μm, the droplets will have an intermediate behavior, which will depend on their intrinsic properties and their environment, especially due to the evaporation mechanism. For droplets of saliva, which contain non‐volatile material, the evaporation mechanism is slower. A decrease in size during 3–4 min, followed by a stabilization at about half the initial droplet diameter, was observed experimentally with saliva droplets.^9^

Simulations of the propagation of respiratory and aerosol droplets generated by speech over a wide range of temperatures (0–40°C) and relative humidity (0–92%) show that 95% of the droplets are deposited over a distance of less than 1.4 m.^10^ However, some droplets may travel greater distances depending on the ambient conditions or initial situation, such as initial ejection speed and entrainment by the turbulent gas cloud when sneezing. The simulation of these phenomena shows that the distance traveled by droplets is less than 1 m for an air movement of 1 m s^−1^ (normal speech), whereas it can pass to 6 m, in the case of an air movement of 50 m s^−1^.^11^ These simulations are consistent with experimental tests, which show that droplets and aerosols produced by coughing and sneezing can reach distances of 7–8 m.^12^ In a simulated classroom, CFD computation shows that a significant fraction (24%–50%) of particles smaller than 15 µm follow the airflow and are extracted within 15 min by the air conditioning system, while particles larger than 20 *µm* tend to settle on the floor, offices, and neighboring surfaces.^13^

The theoretical transport capacity of a spherical droplet depends on its volume, which is proportional to the cube of its radius. It is therefore possible to estimate, for a given viral load scenario, the probability of finding the virus in a droplet or aerosol immediately after emission. Analysis of oral fluid from infected patients shows viral RNA loads ranging from 10^6^ to 10^9^ copies ml^−1^ with a maximum of up to 10^11^ copies ml^−1^.^14^, ^15^ As reported by Stadnytskyi, for a viral load 7 × 10^6^, there is a 37% probability that a droplet of 50 μm (before dehydration) contains at least one virus.^16^

There are numerous field studies showing the presence of SARS‐CoV‐2 or similar viruses in aerosols. These studies have mostly been conducted in hospital settings where COVID patients are concentrated.^17^, ^18^, ^19^, ^20^, ^21^, ^22^ However, other areas have also been investigated and airborne contaminations have been found in public places such as department store entrances^18^ or in urban areas.^23^ Most of these studies were conducted by PCR however, which is not a demonstration per se of the presence of a viable virus. The number of studies investigating the viability of SARS‐CoV‐2 or similar viruses (SARS‐CoV‐1, MERS‐CoV) in air samples is scarce. Of the 11 studies identified by da Silva,^24^ 7 had found positive viability, one had negative viability, and two were reported as uncertain results. One experimental study compared specifically the viability of SARS‐CoV‐2 and SARS‐CoV‐1 in aerosols (< 5 μm). The median half‐lives observed for these two viruses were 1.1–1.2 h respectively.^25^ Similar results were obtained by Smither, who reported half‐lives for SARS‐CoV‐2 in aerosols of artificial saliva of 30–177 min, depending on the conditions of the experiment.^26^

In this study, the hypothesis of aerosol contamination in a poorly ventilated space is addressed through the case of a SARS‐CoV‐2 cluster occurring in a courtroom.

### 1.1 Case description

In October 2020, Unisanté's occupational physicians were called upon by a Vaud state department following the appearance of symptoms compatible with COVID for 5 of the 10 people who participated in a hearing in the same courtroom. The situation was investigated to highlight the course of the hearing, the protective measures put in place, and the ventilation conditions in the courtroom.

The hearing took place behind closed doors on the afternoon of 30.09.2020 from 2 to 5 p.m. in a courtroom belonging to the Canton of Vaud (Switzerland). As this was a formal event, the records of the hearing made it possible to retrace the positions and activities of the participants quite precisely. Nine people, identified P1 to P9, attended the entire hearing and a 10th person (witness, P10) attended the hearing for only 34 min.

**FIGURE 1:**
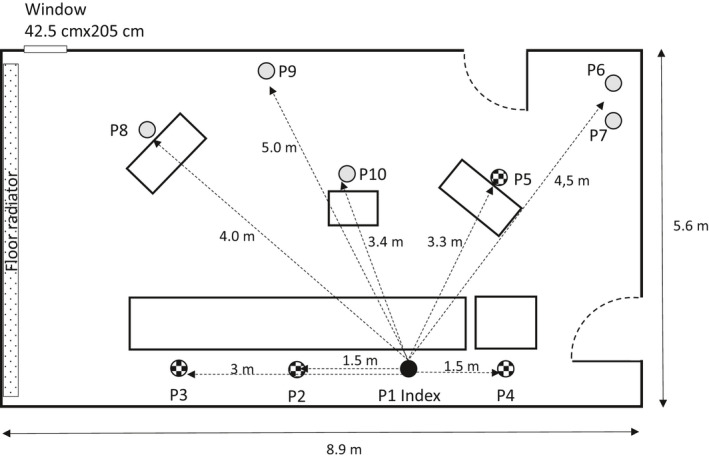
Schematic view of the courtroom and location of the participants during the hearing

P1 arrived alone by car. The hearing started at 2 p.m. in the presence of P1, P2, P3, and P4. From 2:05 p.m., P5, P6, P7, P8, and P9 were also present in the room. The respective positions of the participants in the hearing room are shown in Figure 1. Each participant was assigned a specific seat, with a minimum distance of 1.5 m between each seat. Only P6 and P7, who shared a common household, were seated within 1.5 m of each other. During the session, there was no exchange of seats, as everyone remained seated in the assigned position. There were three breaks during the hearing: a first break from 2:23 p.m. to 2:30 p.m., a second break from 2:55 p.m. to 3:10 p.m., and a third break from 3:44 p.m. to 3:50 p.m. During the breaks P1, P2, P3, and P4 remained in the room, except perhaps to go to the toilet (information unavailable). The five other people (P5, P6, P7, P8, P9) went out to the waiting area in the hall. Witness P10 was present in the courtroom from 3:10 p.m. to 3:44 p.m. and was heard at that time. The hearing ended at 5:00 p.m.

During the hearing, the people did not have a microphone and the exchanges took place in an oratory mode. According to the chairperson, during the hearing, P2, P8, and P5 were the 3 people who spoke the longest and loudest. P1 (index case) and P4 spoke very little. P1, P2, P3, and P4 conversed during breaks, but in “normal” voice. The exchange of objects between participants was very limited. A paper document circulated between P8, P2, P1, and P4. P3 also walked up to P5 and P8 to have them sign a document during the hearing.

At the time of the hearing, Switzerland was at the beginning of the second epidemic wave. The 14 days incidence of cases in the country was about 100 cases/100 000 inhabitants. General hygiene measures and the wearing of masks in closed public places and a social distance of at least 1.5 m were in force. The wearing of a face mask was mandatory inside the building, except when individuals were seated in their assigned seats in the courtroom. Participants therefore removed their masks once they were seated at least 1.5 m apart. Disinfectant was available for hand washing. The participants have, in principle, complied with the existing protective measures.

Examination of the ventilation conditions during the hearing showed that the room was poorly ventilated. The building's logistics department recorded a breakdown of the ventilation system from 28.09.2020 to 6.10.2020. This failure was not known to the participants at the time of the hearing, and therefore during the 3 h of the hearing, the mechanical ventilation did not work. The courtroom was equipped with a narrow window of 42 × 205 cm (width × height). During the hearing, the door and window were always kept closed for confidentiality reasons. But the window was opened during the breaks. Based on weather data, the average daily relative humidity (outdoors) on the day of the hearing was 93.5%. The indoor relative humidity at the time was not known.

**FIGURE 2:**
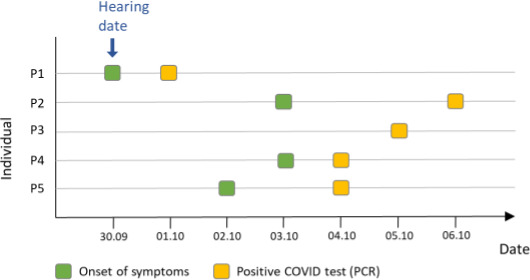
Symptom and test schedule for index case and all four probable secondary cases

The contact tracing carried out afterward by the public health service established that:

- P1 can be considered as the index case and that P2, P3, P4, and possibly P5 are likely secondary cases following the hearing.
- Except for P5, who had a contact with a positive case prior to the hearing, contact tracing did not identify other risk situations for secondary cases (bar, parties, occupational or private exposure). In particular, P2, P3, and P4 had no contact with P1 outside of the hearing and no contact with other COVID cases during the window of contagiousness.
- The index case (P1) presented cough symptoms from 30.09 with a positive test on 1.10. All secondary cases were tested positive within the next week (see Figure 2).

## 2 MATERIAL AND METHODS

### 2.1 Analysis of transmission mode

At the temporal level, the hearing took place on 30.09 with a symptomatic index case on 30.09 and tested positive on 1.10. P2 and P4 reported symptoms on 3.10, i.e., on the 3rd day after contact while P3 onset of symptoms was unknown. P5 mentioned having possibly been exposed to a case at a party on 26.09 and reports symptoms on 2.10, i.e., only 2 days after the hearing. The SARS‐CoV‐2 virus has an average incubation period of 3–7 days and extremes of 2–14 days. The symptoms and positive tests are therefore consistent with the incubation period of the SARS‐CoV‐2. P2, P3, and P4 can reasonably be considered secondary cases to P1. The situation is less conclusive for P5, which endured a very short incubation period and reported having been in contact with a positive case prior to the hearing.

Although the number of participants and contaminations related to this event is quite modest, the interest of this case lies in the precise temporal and situational information obtained on the hearing. This information was supplemented with the contact tracing data for the index case. The analysis of these elements suggests that the aerosol transmission route may have played a determining role in this situation.

- During the hearing, the persons present remained seated in their assigned seats, there was no sharing of seats, and only a paper document was circulated between 3 persons, therefore transmission of the virus by contact with a contaminated surface or object does not seem credible to us. Moreover, the instructions for cleaning the surfaces were respected a priori. The work surfaces of the room's furniture can therefore be considered as clean before the hearing.
- Droplet transmission alone seems unconvincing because the index case spoke very little during the hearing and does not seem to have spoken loudly during breaks. Subsequent measurements in the room showed that the spacing between the chairs of the index case and P4, P2, P3, and P5 were 1.5, 1.5, 3, and 3.3 meters, respectively.
- The hearing lasted 3 hours in a room, which, apart from short breaks, had closed windows and no mechanical ventilation. In the absence of sufficient ventilation, the aerosols generated by the breathing and speech of the participants will concentrate in the room and contribute to increase the dose of quanta received.

The index case sample (nasal swab) was no longer available at the time of this evaluation. However, it was possible to retrospectively analyze the sample from a secondary case, which tested negative for S‐gene dropout. It can be concluded that the cluster is not due to the UK variant.

### 2.2 Field Measurements

Field measurements were conducted on December 21, 2020, in the absence of any hearing in the courtroom. Compared to the studied situation, the only difference during this field measurement was the presence of plexiglas™ dividers (65 × 110 cm) between each seat.

The air renewal was measured by injecting CO_2_ from a pressurized cylinder into the courtroom, until a concentration of about 2200 ppm was reached, the ventilation system being stopped to mimic the conditions at the time of the hearing. CO_2_ was measured using a direct reading CO_2_ sensor (Testo 435, Testo, Mönschaltorf, Switzerland), located on a table in the middle of the room (P10). Two ventilators were in function during the measurement in order to homogenize the ambient air. Once injected, the decaying CO_2_ level in the room was recorded during about 30 min in absence of any person. CO_2_ measurements were repeated with the window open, to mimic the air renewal conditions during the breaks. The air renewal rate was obtained by plotting the natural logarithm of the CO_2_ levels as a function of the time and computing the slope of the linear part of the plot.

The aerosol dissemination within the room was investigated using an ultrasonic atomizer system (25 ml solution h^−1^) filled with a lactose solution (0.1 M in water). The generator was located in P1, mimicking the emission of the index case. Air sampling was performed at all positions (P1–P10), using sampling pumps (SKC AirChek 500; Eighty Four, PA, USA) operating at a flow rate of 2 liter per minute and connected to a sampling open‐face cassette containing a cleaned quartz microfiber filter (Fioroni; 37 mm). Nobody was present in the room once the generation system and the pumps were started. The generation and sampling lasted about 80 min. The aerosol characterization was done by installing a direct reading aerosol spectrometer (Grimm, EDM 109) at P1 and P3. After sampling, the filters were extracted in water and this solution was analyzed for lactose using an ICS‐5000 ion chromatography system (Thermo‐Dionex, USA) equipped with a DP 5000 pump, an autosampler AS‐AP, and a thermostatized compartment DC 500 with an electrochemical detector. Lactose aerosols measurement was chosen because of the submicronic size distribution as well as sensitivity and reliability of lactose determination by ion chromatography.

### 2.3 Modeling viral transmission through aerosols

The conditions of use of the courtroom being known, it is possible to model the exposure situation to estimate an emission rate and analyze the influence of the exposure parameters. The approach used in this study is similar to that proposed by Miller et al.^27^ The probability of infection when inhaling viruses can be described by the Wells‐Riley equation:

(1) $$p=1‐e^{‐n}\left[\%\right]$$

The probability of infection (*p*) is an exponential function of the number of inhaled virus quanta (n). The term quanta is used in evaluating airborne infections, conveying information on both virulence and quantity of infection material. A quanta is the dose of aerosol required to cause infection in 63% of susceptible persons in a room. The formulation of Equ.1 is based on several underlying assumptions: (a) the infectious individual emits SARS‐CoV‐2 at a constant rate, (b) the initial airborne concentration of virus is zero, (c) the latency time of the disease is longer than the time scale of the event, (d) the infectious aerosol is homogeneously distributed in the ambient air, and (e) viruses are eliminated, in a first‐order decrease, by a combination of ventilation, deposition on surfaces, and inactivation. Assumptions a, b, and c appear relatively trivial when considering the moderate variability of aerosol emission in the same activity (normal speech) and the duration of the event relative to the latency time. Hypotheses d and e are less trivial, but are common assumptions in the field of aeraulic. An ideal mixing is for instance difficult to achieve in practice, since the geometry of the room or the presence of obstacles (e.g., furniture) will lead to the creation of short circuits or poorly ventilated areas (dead zones). This model is however commonly used to simplify an overly complex physical situation and obtain an estimate with reasonable resources. Moreover, experimental measurement of the air change rate allows the model to be verified and adjusted.

The number of inhaled quanta depends on the mean aerosol concentration *C_avg_* [q m^−^ *^3^*] and the volume of inhaled air, which in turn depends on the duration of the event D [h] and the breathing rate *Q_b_* [m^3^ h^−1^]. Equation (1) becomes:

(2) $$p=1‐e^{\left(‐C_{\mathrm{avg}}\cdot D\cdot Q_{b}\right)}\left[\%\right]$$

In the case where the probability of transmission is known, the average concentration over the period can be expressed as:

(3) $$C_{\mathrm{avg}}=\frac{‐\ln\left(1‐p\right)}{D\cdot Q_{b}}\left[\text{quanta}\;m^{‐3}\right]$$

The concentration of pollutant emitted by a constant source in an ideally mixed volume is obtained by integrating its material balance equation.^28^ It depends on the quantum emission rate *E* [q h^−1^], the volume of the room *V* [m^3^], the time *t* [h], and the leakage coefficient λ[h^−1^]. The global leakage coefficient of the virus is itself dependent on the deposition on the surfaces λ_dep_ [h^−1^], the air renewal λ_v_ [h^−1^], and the virus decomposition k [h^−1^].

(4) $$C\left(t\right)=\frac{E}{\lambda \cdot V}\left(1‐e^{‐\lambda \cdot t}\right)\left[\text{quanta}\;m^{‐3}\right]$$

$$\text{with}\;\lambda =\lambda_{\mathrm{dep}}+\lambda_{v}+k$$

The integration of Equation (4) over the exposure time D allows expressing the average concentration in the compartment assuming a constant emission, no initial concentration in the room, and a negligible external concentration.

(5) $$C_{\mathrm{Avg}}=\frac{E}{\lambda \cdot V}\left[1‐\frac{1}{\lambda \cdot D}\cdot \left(1‐e^{‐\lambda \cdot D}\right)\right]\left[\text{quanta}\;m^{‐3}\right]$$

Using Equations (2) and (5), the emission rate can be estimated using the probability of infection *p* and the environmental exposure conditions in the courtroom.

### 2.4 Implementation

The calculations have been implemented on Stata/IC 16.1 (StataCorp LLC, TX, USA). Table 2 summarizes the parameters used in the model. Since some parameters are not precisely known, random sampling was carried out considering the possible distribution of values (*n* = 1 000 000). To facilitate the comparison of results, the parameters used are compared with those proposed by Miller for the Skagit Valley situation.^27^

**TABLE 2:** Parameters used in the simulation for estimating the emission rate E, comparing values between Miller et al. (Skagit Valley Event) and our case (Courtroom Event)

Parameter	Value (Skagit Valley)	Value (Courtroom)	Distribution	Source
Probability of infection, p [%]	53–87	33–44	Uniform	Reported attack rate^36^
Breathing rate Q_b_ [m^3^ h^−1^]	0.65–1.38	0.32–0.76	Uniform	^37^
Loss rate due to ventilation, λ_v_ [h^−1^]	0.3–1.0	0.23 (closed windows)		
1.25 (open windows)	Uniform	Measured value		
Loss rate due to surface deposition, λ_dep_ [h^−1^]	0.3–1.5	0.3–1.5	Uniform	^38^
Loss rate due to virus inactivation, k [h^−1^]	0–0.63	0.26–1.08	Uniform	^25^
Room volume, V [m^3^]	810	150	Constant	Measured value
Duration of exposure [h]	2.5	3	Constant	Reported values (hearing's minutes)

A transmission probability (secondary attack rate) of 33%–44% (3/9–4/9) was used for the courtroom situation. Without considering the index case, 4 of the 9 people present were indeed confirmed SARS‐CoV‐2 positive within days of the hearing. For one of the cases (P5), however, contamination by the index case is not very likely due to the short incubation period and prior contact with a positive case at a party. The most likely value is therefore 3/9 with an upper estimate of 4/9.

The air renewal measured in the room (loss rate to ventilation λv) was of 0.23 h^−1^with the window closed and of 1.25 h^−1^ with the window open. Since it is difficult to know if the window was open during all the breaks, a uniform distribution ranging between these two values was used for λ_v_.

Volumetric inhalation rates were taken from the Binazzi study. The value used here is that of "reading aloud with a normal voice", which is lower than that of singing. Doremalen's study was selected to determine a plausible range of loss rate due to virus inactivation. The half‐life interval proposed by this study is 0.64–2.64 h, corresponding to a k of 0.26–1.08 h^−1^.

## 3 RESULTS

**FIGURE 3:**
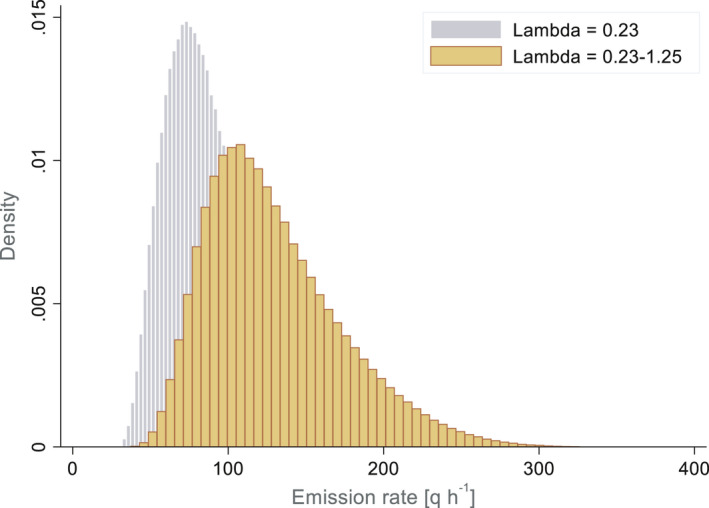
Estimated distribution of the emission rate in the courtroom situation

The probable distribution of the resulting emission rate is shown in Figure 3. The mean value is 130 q h^−1^ with an interquartile (25–75 percentile) of 97–155 q h^−1^ and a 50th percentile of 122 q h^−1^. To evaluate the effect of our hypothesis on the distribution of the loss rate due to ventilation, a simulation was also carried out for a closed window for the duration of the hearing (λ_v_ = 0.23). In this more conservative case, an average emission rate of 90 q h^−1^ is sufficient to produce the same attack rate. The emission rate remains in the same order of magnitude, but the distribution of the emission rate is significantly narrower, reflecting the smaller range of uncertainty used on the input parameters.

The investigations conducted, by lactose emission, allowed a better understanding of the distribution of aerosols inside the room. The results of the sampling carried out are presented in Table 3. Apart from the concentration at location P1, which corresponds to the position of the emission source, the concentrations in the different locations (P2‐P10) are relatively homogeneous (*n* = 8, mean 336 µgm^−3^, SD = 39 µgm^−3^; interquartile range 315–344 µgm^−3^). Even with the presence of plexiglas™ (added after the hearing), which tends to limit horizontal airflows, these concentrations remain in the same order of magnitude, suggesting that the well‐mixed model used is adequate.

**TABLE 3:** Lactose concentration observed in the room at different locations, with an emission source at P1

Position in the room	Conc. Lactose [µg.m^−3^]
P1 (emission source)	1910
P2	423
P3	344
P4	347
P5	325
P6‐7	329
P8	292
P9^(a)^	310
P10	317

Note

^(a)^ approximate position of P9, based on seating plan.

Examination of the direct reading measurements made at locations P1 and P3 shows differences in the aerosol size distribution (see Supplementary material). At P1, the generated lactose aerosol presented a mass‐based size distribution with a geometric mean diameter of 0.63 µm (GSD 1.72 µm), in agreement with the mean size of exhaled aerosols (0.7–1 µm),^29^ while at P3, the mean diameter measured was 1.45 µm (GSD 1.82). These results suggest that aggregation or coagulation mechanisms take place, such as the adsorption of viruses on particulate matter (PM),^30^ in which the particles became the “carriers” of SARS‐CoV‐2.

**FIGURE 4:**
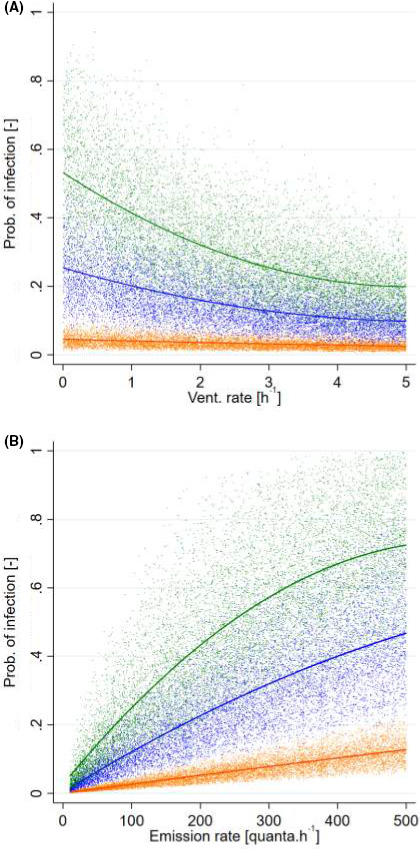
(A) Probability of infection simulated in a 150 m^3^ room for various loss rate due to ventilation (λ_v_) and event duration: orange: 0.5 h, blue 1.5 h, green 3 h (*n* = 30 000). (4B) Probability of infection simulated in a 150 m3 room for various emission rates E and event duration: orange: 0.5 h, blue 1.5 h, green 3 h (*n* = 30 000)

An exposure scenario was considered to illustrate the influence of environmental and emitter‐related parameters (the index case) on the contamination conditions in the room. The model of the well‐mixed room, previously described in Equations (4) and (5), was used to calculate the probability of contamination in the courtroom, with a variable air exchange rate, ranging between 0 and 5 h^−1^ and three possible event durations: 0.5, 1.5, and 3 h. The distributions used for the simulation parameters are those already described in Table 3. The effect of the loss rate related to ventilation (λ_v_) is shown in Figure 4a. Despite a strong dispersion, linked to the distributions of the input parameters, in particular that of the previously calculated emission rate, it shows that the renewal rate plays an important role for event durations greater than 0.5 h. Its effect is however moderate beyond an air change of 2–3 h^−1^. The effect of the emission rate E is shown in Figure 4b.

Unsurprisingly, the probability of infection is strongly influenced by the release of the virus. Again, this influence is relatively modest for relatively short event times (0.5 h or less). For longer event times, a rapid increase is observed between 0 and 300 q h^−1^. For higher emission rates and event times, this growth is attenuated, probably due to the exponential nature of the probability of contamination (see equation 2).

## 4 DISCUSSION AND CONCLUSION

Existing knowledge on the aeraulic behavior of fine particles, speech and respiration‐related emissions, and experimental evidence on the viability of the virus in the air support a contribution of SARS‐CoV‐2 transmission by inhalation of aerosols. However, its identification in situ is difficult, probably because it is often not possible to distinguish the relative contribution of the different transmission routes. This study contributes to the modest but growing body of case studies highlighting this mode of transmission. Compared to other similar studies, the cluster (*n* = 5) investigated in this study is small in size, which makes the estimation of the attack rate (probability of secondary infection) uncertain. It cannot be excluded that some contamination may have occurred outside of this situation. On the other hand, it is possible that individuals who would have already been contaminated during the first spring wave, but who were asymptomatic, were present in the courtroom and benefited from some immunity. Despite of these uncertainties, the main interest of this case is that the conditions of the courtroom hearing were well known and documented, which allows us to reasonably exclude that the contaminations observed could have been explained by other transmission modes.

The emission rate distribution obtained in this study has a mean value of 1.3 × 10^2^ q h^−1^. This is significantly lower than the estimated value in the case of the Skagit Valley, which is 9.7 × 10^2^ q h^−1^. This difference can be explained by different environmental conditions in the room (volume, renewal rate), but also by the fact that Miller et al. made conservative assumptions. In particular, they hypothesized that inhalation of aerosols was the dominant form of contamination, whereas it is likely that some of the 53 identified cases were contaminated by contact or droplets. The values we estimated for the courtroom situation are close to the upper emission range estimated by Buonanno et al.^14^ For individuals sitting or standing without physical exercise and busy talking, they proposed emission rates of 10^2^ q h^−1^ in individuals with elevated viral loads and assuming a high infectivity coefficient (c_i_). This value of emission rate corresponds, for example, to a viral load of 10^9^ RNA copies ml^−1^ for a c_i_ of 0.1.

Analysis of the influence of event duration, emission rate, and ventilation loss rate highlights the importance of these parameters in the indoor transmission of SARS‐CoV‐2. On the one hand, situations with a low theoretical infection probability are mainly associated with emission rates below 100 q h^−1^ and relatively short event durations (typically 0.5 h). For these situations, the ventilation loss rate plays only a marginal role in transmission. On the other hand, high infection probabilities (e.g., >30%), which may lead to large infectious clusters, are due to the combination of several unfavorable parameters, generally requiring emission rates greater than 100 q h^−1^, ventilation rates <2 h^−1^_,_ and event durations equal to or greater than 1.5 h. This is consistent with the cases of super propagation reported in the literature and associated with aerosols, which are surprisingly few in number and highlight situations of confinement, insufficient ventilation, and/or high emissions rates.^27^, ^31^, ^32^ Improving air renewal conditions in meeting spaces reduces the risk of transmission in general. However, our results suggest that an effective strategy to combat large infectious clusters would be to target “hot spots”, where groups of individuals spend several hours in poorly ventilated spaces, as a priority.

The example given here is that of a 150 m^3^ room and the results obtained are not readily transposable to larger or smaller volumes such as conference rooms or domestic rooms. It is interesting to note that 150 m^3^ is typically in the order of magnitude of the volume of classrooms, the ventilation of which is a matter of debate. Our results suggest that while room ventilation is essential, it is difficult to control the risk of contamination with this parameter alone because of the residual probability of infection at high ventilation rates, brought by the variability of the other parameters (e.g., duration of exposure and emission rate). Keeping in mind that the number of transmitters and targets also increases linearly with the number of people in the room. Wearing a community or surgical mask, which contributes to mitigate emission at source, especially during voice bursts, coughing, or sneezing, seems essential.

## 5 AUTHOR CONTRIBUTORS

DV and SS planned the study. JJS, DV, and GS planned the field investigations. SS and CP investigated the case study JJS, and GS conducted field investigations. JJS, GS, and DV conducted the data analysis, DV did the modeling and wrote the first draft. All co‐authors contributed to the revision and finalization of the manuscript. The corresponding author attests that all listed authors meet authorship criteria and that no others meeting the criteria have been omitted.

## FUNDING INFORMATION

The author received no specific funding for this work.

## CONFLICT OF INTEREST

No conflict of interest.

### PEER REVIEW

The peer review history for this article is available at https://publons.com/publon/10.1111/ina.12866.

## Supporting information

Supplementary Material

## Data Availability

Detailed primary and secondary data used for this study are available upon request.
