# Supplementary material for: Probable aerosol transmission of SARS‐CoV‐2 in a poorly ventilated courtroom
Source: Indoor Air. 2021 Jun 11;31(6):1776–85. doi: 10.1111/ina.12866 (PMC8597151; doi:10.1111/ina.12866)
Supplement: Supplementary file 1 — Supplementary Material [file INA-31-1776-s001.docx]

Supplementary material

*Figure S1. Size distribution of the aerosol generated at the location of the Index case (P1), using lactose, and the corresponding size distribution recorded at a distance of 3 meters (P3).*
